# Prophylactic Treatment with Hydrogen Sulphide Can Prevent Renal Ischemia-Reperfusion Injury in L-NAME Induced Hypertensive Rats with Cisplatin-Induced Acute Renal Failure

**DOI:** 10.3390/life12111819

**Published:** 2022-11-08

**Authors:** Ashfaq Ahmad

**Affiliations:** Department of Pharmacy Practice, College of Pharmacy, University of Hafr Al Batin, Hafr Al Batin 39524, Saudi Arabia; ashfaqa@uhb.edu.sa; Tel.: +96-65-0430-9874

**Keywords:** hydrogen sulphide, cisplatin, renal failure, nuclear factor kappa B, inter cellular adhesion molecule, non-renal failure, ischemia-reperfusion injury

## Abstract

(Background and Objectives): Renal ischemia perfusion injury is one of the major issues in kidney transplant. The aim of the study was to investigate the hypothesis that prophylactic treatment—with a hydrogen sulphide donor to an acute renal failure case of hypertensive rats—can minimize the ischemia reperfusion injury of the kidney which is beneficial for kidney transplant. To check this hypothesis, the present study was designed to investigate the effect of chronic administration of a hydrogen sulphide (H_2_S) donor and sodium hydrosulfide (NaHS) on nuclear factor kappa B (NF-kB) and inter cellular adhesion molecule-1 (ICAM-1) concentration in non-renal failure (NRF) and acute renal failure (ARF) rats in the ischemia-reperfusion injury (IRI) model of the kidney in both normotensive WKY and hypertensive rats (L-nitro arginine methyl ester (L-NAME-induced); (Materials and Methods): A total number of 48 Sprague-Dawley rats were recruited into eight groups each consisting of six animals. Each of these eight groups was used to measure systemic and renal parameters, H_2_S, antioxidant parameters in plasma, plasma concentration of NF-kB and ICAM-1 and renal cortical blood pressure. ARF was induced by single intraperitoneal (i.p.) cisplatin injection (5 mg/kg). Hypertension was induced by oral administration of L-NAME in drinking water for four weeks at 40 mg/kg/day. NaHS was administered (i.p) at 56 µmol/kg for five weeks while dL-propargylglycine (PAG), a H_2_S generation inhibitor, was administered as a single intra-peritoneal injection (50 mg/kg). An acute surgical experiment was performed for the induction of renal ischemia for 30 min by renal artery clamping followed by reperfusion for three hours; (Results): Chronic administration of NaHS attenuated the severity of ARF in both normotensive and hypertensive animals (L-NAME) along with lowering the blood pressure in hypertensive groups. NaHS improved the oxidative stress parameters such as total superoxide dismutase (T-SOD), glutathione (GSH) and reduced the malondialdehyde (MDA) concentration along with reduction of NF-kB and ICAM-1 following renal IRI; Conclusions: These findings demonstrate that H_2_S not only reduced the severity of cisplatin induced ARF but also reduced the severity of renal IRI by upregulating antioxidants along with decreased concentrations of NF-kB and ICAM-1 in normotensive and L-NAME induced hypertensive rats.

## 1. Introduction

Ischemia-reperfusion injury (IRI) impairs the function of a reperfused organ and is always a challenge for kidney transplantation. IRI injury results in increased production of reactive oxygen species (ROS) which induces inflammatory responses and in turn results in leukocytes adhesion, rolling and their transmigration to the ischemic tissues after reperfusion [1]. Intercellular adhesion molecule-1 (ICAM-1) which plays a significant role in inflammatory process, serves as a carrier for the diapedesis of leukocytes to the target zone and is expressed on the surface of leukocytes [2] as well as on the endothelium [3]. ICAM-1 play a crucial role in potentiating inflammatory responses [4] and these responses cause post-ischemic organ injury. Nuclear factor kappa B (NF-kB) which is involved in the expression of inflammation and the expression of inflammatory genes, controls the transcription of the ICAM-1 gene [5] and lies in non-stimulated cells in an inactive form in the cytoplasm along with their inhibitory kappa B (IkB) subunits. Once IkB is degraded by any stimulus, it causes the entrance of NF-kB from the cytoplasm into the nucleus where it induces its specific mRNA synthesis. Transcription factor NF-kB controls the expression of various specific genes and its activation by an appropriate stimulus may elevate the intensity of IRI [6]. Production of ROS stimulates NF-kB activation [7] and this activation in response induces the expression of ICAM-1 [8]. Altogether, inflammatory responses and the production of ROS play a crucial role in the pathophysiology of IRI via NF-kB and the ICAM-1 pathway.

Different treatment approaches in the laboratory animals have been studied to minimize the severity of IRI by taking inflammation and oxidative stress into consideration. Treatments including ROS scavengers such as antioxidant therapies such as catechin [9] and naringin [10] have been previously reported to be beneficial in reducing the degree of IRI. H_2_S has anti-inflammatory properties [11] along with antioxidant potential [12,13], therefore due to the involvement of oxidative stress and inflammation in IRI, the present study was designed to explore the beneficial effects of long-term pre-conditioning with H_2_S.

Hydrogen sulphide (H_2_S) has been classified as a third gasotransmitter [14] after carbon monoxide (CO) and nitric oxide (NO). It is an important intercellular and intracellular gaseous messenger molecule [15]. Hydrogen sulphide is produced inside the human body by following three pathways involving L-methionine, L-cysteine and 3-Mercaptopyruvate by involving different enzymatic pathways. Among these three pathways, L-methionine involves one enzyme cystathionine-β-synthase (CBS) while L-cysteine involves two enzymes cystathionine-γ-lyase (CSE) and CBS to produce H_2_S in the body [16,17,18,19]. CBS is mainly responsible for producing H_2_S in the brain, while CSE mainly pre-dominates its production in the heart [20,21] and kidneys [22]. Recently, 3-mercaptopyruvate sulphurtransferase (3-MST) in combination with cysteine aminotransferase (CAT) has been reported to be responsible for approximately 90% production of H_2_S in the brain [20,23]. Various physiological properties of H_2_S such as anti-hypertensive [24,25,26], vasorelaxant [27,28,29], anti-inflammatory [30] and antioxidant potential [12,31] have been reported in the past. Hydrogen sulphide (H_2_S) has anti-inflammatory properties [11] along with antioxidant potential [12,13]. Therefore, due to the involvement of oxidative stress and inflammation in IRI, the present study was designed to explore the beneficial effects of long-term pre-conditioning with H_2_S.

Reno-protective effect of NaHS (H_2_S donor) either topically [32] or its flushing through the kidneys [33,34] have been evident in normotensive model of renal IRI. At present, the effect of chronic exogenous administration of NaHS in normotensive and hypertensive models of acute renal failure (ARF) in IRI is unknown. Current study explored that prophylactic treatment of NaHS (five weeks) on renal excretory function in normotensive rats with ARF and L-NAME induced hypertensive rats with ARF following renal IRI. The current study investigated ARF model because ARF is characterized by decreased endogenous concentration of H_2_S [35] and by increased oxidative stress [36]. Similarly, increased oxidative stress [37] and decreased endogenous concentration of H_2_S [26] has been reported in L-NAME induced hypertensive rats. It was expected that the kidneys of L-NAME induced hypertensive and ARF rats are at higher risk to the consequences of IRI when compared to that of normotensive rats. 

The aim of the study is to investigate the hypothesis that prophylactic treatment with H_2_S donor to an acute renal failure case of hypertensive rats can minimize the ischemia reperfusion injury of the kidney which is beneficial for kidney transplant. To check this hypothesis, the present study was designed to investigate the effect of chronic administration of H_2_S on NF-kB and ICAM-1 concentration in non-renal failure (NRF) and acute renal failure (ARF) rats in the IRI model of the kidney in both normotensive WKY and hypertensive rats (L-NAME-induced)

## 2. Materials and Methods

### 2.1. Chemicals and Drugs

L-NAME was purchased from China (Sigma-Aldrich, Shanghai, China), NaHS (Sigma-Aldrich, St. Louis, MO, USA), PAG (Sigma-Aldrich, Schaffhausen, Switzerland), Cisplatin (United Pharm., Inc., Seoul, Korea) and pentobarbital sodium (Dorminal 20%, Alfasan, Woerden, The Netherlands).

### 2.2. Ethical Statement and Experimental Groups

The present study was conducted with approval from the Animal Ethics Committee of University Sains Malaysia (AECUSM) with approval reference no./2015/(95) (649). Forty-eight male Wistar Kyoto (WKY) rats weighing 225 ± 15 gm were secured from theAnimal Research and Service Centre, University Sains Malaysia (ARASC, USM) and were permanently shifted for study duration to the localized facility for the animals in theSchool of Pharmaceutical Sciences, USM for acclimatization. During the study period, all the animals in the transit room were given water and standard rat chow (Gold Coin. Sdn, Bhd, Selangor, Malaysia) throughout the study period time. The present study included two main study groups: WKY and L-NAME. Both groups were further subdivided into four sub-groups (n = 6) consisting of (1) groups that received only saline (WKY-CONTROL and L-NAME-CONTROL), (2) groups that received saline plus cisplatin injection (inj.) for the induction of ARF (WKY-RF and L-NAME-RF), (3) groups that received NaHS and cisplatin inj. (WKY-RF+NaHS and L-NAME-RF+NaHS) and (4) PAG and cisplatin injection groups (WKY-RF+PAG and L-NAME-RF+PAG). Details of the study protocol are shown in Figure S1.

### 2.3. Induction of ARF, Hypertension, Adminiistration of NaHS and Metabolic Data Collection

ARF was induced on day 28 of the study period by a single intra peritoneal (i.p.) cisplatin inj. at 5 mg/kg as reported previously [38,39]. Hypertension was induced by the administration of L-NAME (L-nitro methyl argi-nine ester) in drinking water daily for four weeks at 40 mg/kg/day as reported previously [40,41]. L-NAME administration was started on day 8 and continued until day 35 (four weeks) of the study period. L-NAME dose was adjusted after every 3 days according to the body weight of animals. NaHS was administered daily for 35 days i.p. at 56 µmol/kg as reported previously [42]. NaHS was dissolved daily in 0.9% (w/v) normal saline to prepare stock solution and dose was adjusted after every 3 days according to the body weight of animals. CSE inhibitor, (PAG) was injected intraperitoneally as single dose 50 mg/kg on acute experiment day (day 36) and was freshly prepared in 0.9% (w/v) normal saline.

Different parameters such as body weight, water intake (24 h) and urine output were measured on different days (0, 21 and 35) of the study by keeping the rats in the metabolic cages (Nalgene, Thermo Scientific, Rochester, NY, USA). Collection of blood samples was performed from the lateral vein of the rat’s tail on the same days as mentioned above. Plasma samples were separated from the blood by centrifugation for 10 min at 10,000 rpm [43]. Both the urine and plasma samples were kept at −30 °C for future biochemical tests. The kidney index was calculated by using the following formula:

$$\mathrm{Kidney}\;\mathrm{indiex}=\frac{\mathrm{Kidney}\;\mathrm{weight}}{\mathrm{Body}\;\mathrm{weight}}\times 100$$

### 2.4. Measurement of NIBP

Non-invasive blood pressure was measured using CODA (Kent Scientific Corporation, Torrington, CT, USA) by tail cuff method on days 0, 21 and 35. Briefly, rats were acclimatized and trained to stay in a restrainer for 30 min before the actual blood pressure monitoring started. The cuff was placed around the tail and a restrainer was placed on the thermostat which has three levels of heating (L1, L2 and L3). Level 2 was provided for gentle heating to maintain the body temperature of the rats. All the procedures were prefixed including number of cycles, subcycles and interval between each cycle.

### 2.5. Acute Surgical Experiment

An acute surgical experiment was performed as reported previously [43,44], with slight modifications, on day 36 of the study period. Rats were given general anesthesia with 60 mg/kg dose of pentobarbital sodium via i.p. For a clear air passage, tracheotomy was performed with PP 240 tracheal tube. Cannulation of left jugular vein was performed with PP 50 cannula for the administration of maintenance dose (pentobarbital sodium, 12.5 mg/kg). Cannulation of the right carotid artery was also performed with a PP 50 cannula and was connected to a pressure transducer which was linked to PowerLab (ADInstruments, New South Wales, Australia) through a Quad amp (ADInstruments, New South Wales, Australia) to monitor and record systolic B.P (SBP), diastolic B.P (DBP), mean arterial pressure (MAP), heart rate (HR) and pulse pressure (PP). The left iliac artery and left kidney were exposed by a midline abdominal incision. The cannulation of the iliac artery connected to a pressure transducer was performed with a PP 50 cannula linked with the Powerlab (ADInstruments, New South Wales Australia) system for the measurement of iliac B.P. A laser doppler flow probe was placed on the dorsal surface of the kidney to measure renal cortical blood perfusion (RCBP). Once all the surgical procedures were completed, the rat was allowed to stabilize for 1 h followed by baseline values of systemic hemodynamic parameters (SBP, DBP, MAP, HR and PP) and an RCBP recording.

#### 2.5.1. Collection of Urine and Blood Samples

The urinary bladder was catheterized with PP 90 tubing to collect urine samples. Approximately 1.5 mL of blood was collected through the carotid artery cannula. The withdrawn blood was centrifuged for collection of plasma and stored at −30 °C along with the urine sample.

#### 2.5.2. Induction of Ischemia-Reperfusion

Rats were allowed to stabilize for 30 min so that all the variables normalized. After this, renal ischemia was induced by clamping the renal artery by a non-traumatic arterial clamp for 30 min [45]. The ischemic period was followed by 3 h of the reperfusion phase. At the end of the reperfusion phase urine along with a blood sample (for collection of plasma) was collected and stored at −30 °C. The experiment was terminated by euthanizing the rats with pentobarbital sodium (200 mg/kg). After euthanasia, the left kidney was excised, cleared of all connective tissues and the kidney index was calculated using the following formula [43]. 

### 2.6. Measurement of Plasma H_2_S Concentration

The plasma levels of H_2_S were measured on different days (0, 21 and 36) via the spectrophotometric method as reported previously [42,46]. Briefly, the method of measurement of H_2_S involves four steps trapping of H_2_S, processing of samples, precipitation of protein and Centrifugation of the samples.

### 2.7. Renal Function Parameters Measurement 

Potassium and sodium in urine and plasma were measured through flame photometry. From these readings, fractional excretions of sodium (FENa), potassium (FEK), absolute urinary excretions of sodium (UNaV), potassium (UKV) and the urinary sodium to urinary potassium ratio (Na:K ratio) was then calculated. Creatinine concentration in urine and plasma was measured by the colorimetric method as previously reported [47] and these readings were then used to calculate creatinine clearance.

### 2.8. Oxidative Stress Parameters Measurement

Glutathione (GSH), superoxide dismutase (T-SOD) and malondialdehyde (MDA) were measured in plasma in pre-ischemic and reperfusion phases on the acute experiment day (day 36 of the study period) by commercially available kits (NJJC Bio Inc., Nanjing, China) of GSH, T-SOD and MDA.

### 2.9. Measurement of Plasma and Kidney Tissue Concentrations of ICAM-1 and NF-kB 

Plasma concentration of NF-kB and ICAM-1 were measured on the acute experiment day (terminal day of the study) in the pre-ischemia and reperfusion phases. NF-kB concentration was measured quantitatively in the plasma and kidney tissues by using “Rat nuclear fac-tor kappa B ELISA kit” (CUSABIO Biotech Co., Ltd., Houston, TX, USA) having catalog number CSB-E13148r used. Similarly, concentration of ICAM-1 in the plasma was measured quantitatively by using “Quantikine ELISA Rat sICAM-1/CD54” ELISA kits (R&D Systems, Inc. Minneapolis, MN, USA having catalog number RIC100). Instructions given in the kit manuals were followed for ICAM-1 and NF-kB assay procedures. Optical density of all the samples for NF-kB and ICAM-1 were measured at 450 nm using a spectrophotometer (BioTek Instruments, Inc, Winooski, VT, USA). Respective standard curves of known concentrations of ICAM-1 (31.3–1000 pg/mL) and NF-kB (1.56–100 pg/mL) were constructed for the calculations of ICAM-1 and NF-kB measurements.

### 2.10. Histopathology of Kidney by Using Hematoxylin and Eosin (H&E) Staining

Histopathology of the kidney tissues of all groups were done following the procedure reported [20]. Briefly, the histopathology method includes processing of the tissue by using different steps that include sectioning, processing, fixation, dehydration, clearing from alcohol, trimming, sectioning, floating, fixation, and staining with hematoxylin and eosin.

### 2.11. Statistical Analysis 

Data was presented as the mean ± SD and was analyzed by using the statistical software “GraphPad Prism” (GraphPad Prism for windows, version 5.0, Inc., San Diego, CA, USA). A repeated measures one-way ANOVA was used for analysis and comparison of the data which was followed by the Bonferroni post hoc test with significance set at 95% confidence (*p* < 0.05).

## 3. Results

A statistically significant increase in body weight (BW) was observed in all study groups on day 21 when compared to day 0 (Table 1). However, when ARF was induced on day 28, no further increase in BW was observed in RF groups in WKY and L-NAME induced hypertensive rats on day 35. It was observed that the WKY-RF and L-NAME-RF groups had significantly reduced BW on the final day (35th) when compared to the WKY-CONTROL on the same day. Similarly, L-NAME-RF group had significantly reduced BW on the final day (35th) when compared to L-NAME-CONTROL on the same day. Prophylactic treatment with NaHS significantly increased (*p* < 0.05) BW in L-NAME-RF+NaHS on the final day (35th) when compared to L-NAME-RF on the same day. In the present study, it was observed that L-NAME induced hypertensive rats had significantly reduced BW when compared to WKY rats. No significant difference in water intake (WI) was observed in all groups when compared to their respective groups on all days. After the induction of ARF on day 28, a significant increase (all *p* < 0.05) in urine output (UOP) and urine flow rate (UFR) were observed on day 35 in RF groups of WKY and L-NAME treated groups when compared to their respective comparative groups on days 0 and 21. Prophylactic treatment with NaHS significantly reduced (all *p* < 0.05) UOP and UFR on day 35 in WKY and L-NAME treated groups (WKY-RF+NaHS and L-NAME-RF+NaHS) in comparison to their respective untreated groups (WKY-RF and L-NAME-RF) on the same day. It was observed that UOP and UFR were significantly increased (*p* < 0.05) in RF groups of WKY and L-NAME on day 35 in comparison to their respective control of NRF groups of WKY and L-NAME on the same day.

### 3.1. Systemic Haemodynamics

No significant difference in all systemic hemodynamic parameters (SBP, DBP, MAP, HR and PP) was observed in all WKY groups when compared to their respective groups on all days (Table 2). However, a significant increase in SBP, DBP, MAP and HR was observed in all L-NAME groups on day 21 when compared to their same groups on days 0 and 35 in comparison to days 0 and 21. Prophylactic treatment with NaH significantly reduced SBP, DBP and MAP in the L-NAME-RF+NaHS group on day 35 when compared to L-NAME-RF on the same day. It was observed that systemic hemodynamics was significantly higher in L-NAME groups when compared to groups of WKY.

### 3.2. Renal Function Parameters

It was observed that after the induction of ARF on day 28, there was a significant decline (all *p* < 0.05) in plasma sodium, urinary creatinine and creatinine clearance while there was a significant increase (all *p* < 0.05) in urinary sodium, urinary potassium, FE_Na_, FE_K_, U_Na_V and plasma creatinine in the RF groups of WKY and L-NAME rats on day 35 when compared to corresponding groups on days 0 and 21 (Table 3). RF groups of WKY and L-NAME treated rats had significantly increased (all *p* < 0.05) FE_Na_, FE_K_, U_Na_V and plasma creatinine while these groups had significantly decreased urinary creatinine and creatinine clearance on day 35 when compared to their respective NRF control groups (WKY-CONTROL and L-NAME-CONTROL) on the same Prophylactic treatment with NaHS caused significant increase (*p* < 0.05) in plasma sodium in WKY-RF+NaHS while significant decrease in FE_Na_, FE_K_ and U_Na_V in WKY-RF+NaHS and L-NAME-RF+NaHS on day 35 when compared to their corresponding untreated RF groups (WKY-RF and L-NAME-RF) on the same day. It was noticed that L-NAME groups had significantly decreased (*p* < 0.05) plasma sodium, urinary creatinine and creatinine clearance and significantly increased (*p* < 0.05) urinary sodium, urinary potassium, FENa, FEK and plasma creatinine on day 35 when compared to WKY groups on the same day. After the induction of ARF on day 28, UKV was significantly reduced (all *p* < 0.05) on day 35 only in RF groups L-NAME induced hypertensive rats when compared to their corresponding groups on day 0 while the Na:K ratio was reduced significantly (*p* < 0.05) only in the WKY groups on day 35 when compared to their respective groups on days 0 and 21. Prophylactic treatment with NaHS significantly increased UKV in the L-NAME-RF+NaHS group on day 35 when same was compared to the L-NAME-RF group on the same day.

It was noticed that the L-NAME groups had significantly decreased (*p* < 0.05) plasma sodium, urinary creatinine and creatinine clearance and significantly increased (*p* < 0.05) urinary sodium, urinary potassium, FENa, FEK.

#### 3.2.1. Plasma Concentration of H_2_S

**Figure 1:**
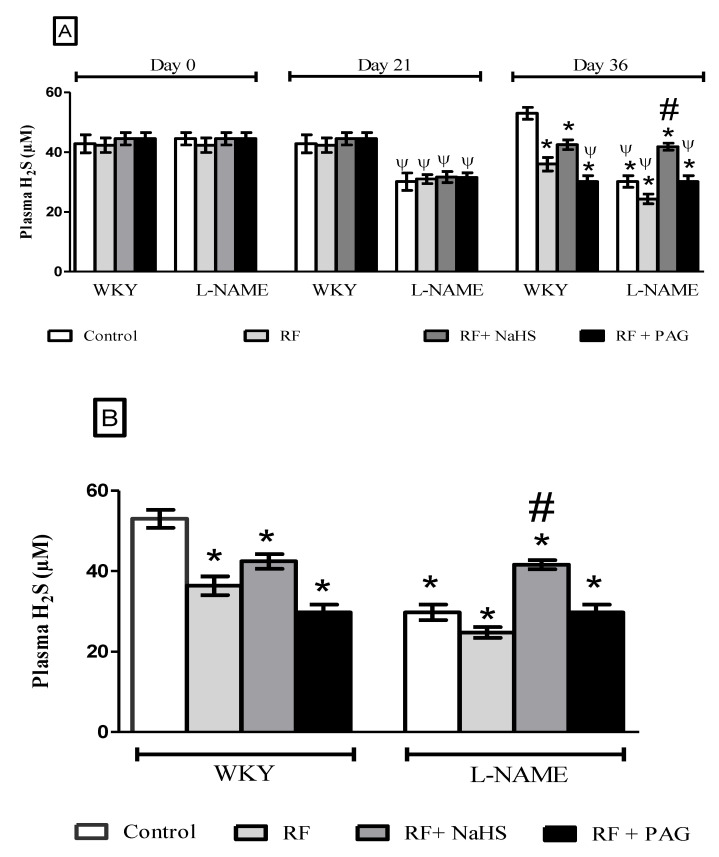
(**A**): Plasma H_2_S concentration on days 0, 21st and 36th (n = 6) while (**B**): Plasma H_2_S concentration on day 36. All the data is presented as mean ± SD with *p* < 0.05. ψ vs. day 0 of respective groups; * vs. WKY-RF on day 36th of the study; # vs. RF on day 36.

It was observed that after the induction of ARF on day 28, concentration of H_2_S in plasma was significantly reduced (all *p* < 0.05) on the acute experiment day (36th day) in the RF groups of WKY and L-NAME induced hypertensive rats (Figure 1A,B). Prophylactic treatment with NaHS significantly increased (*p* < 0.05) plasma concentration of H_2_S on day 36 in the WKY and L-NAME groups which were pre-treated with NaHS (WKY-RF+NaHS and L-NAME-RF+NaHS) when compared to respective untreated RF roups (WKY-RF and L-NAME-RF) and also to PAG administered groups (WKY-RF+PAG and L-NAME-RF+PAG) on the same day. It was noticed that L-NAME induced hypertensive rats as well as RF groups of WKY and L-NAME induced hypertensive rats had a significantly lowered (all *p* < 0.05) H_2_S concentration in plasma as compared to the WKY-CONTROL group on day 36.

#### 3.2.2. Body Weight and Kidney Index on Day 36 (Acute Experiment day)

A significant decline in body weight while a significant increase in kidney index (KI) was observed in the WKY-RF groups and L-NAME groups (WKY-RF and L-NAME-RF) when compared to their NRF control groups as shown in Table 4. KI was significantly higher (*p* < 0.05) in L-NAME-CONTROL groups when it was compared to KI of WKY-CONTROL groups.

#### 3.2.3. Renal Cortical Blood Perfusion in Pre-Ischemia and Reperfusion Phases

**Figure 2:**
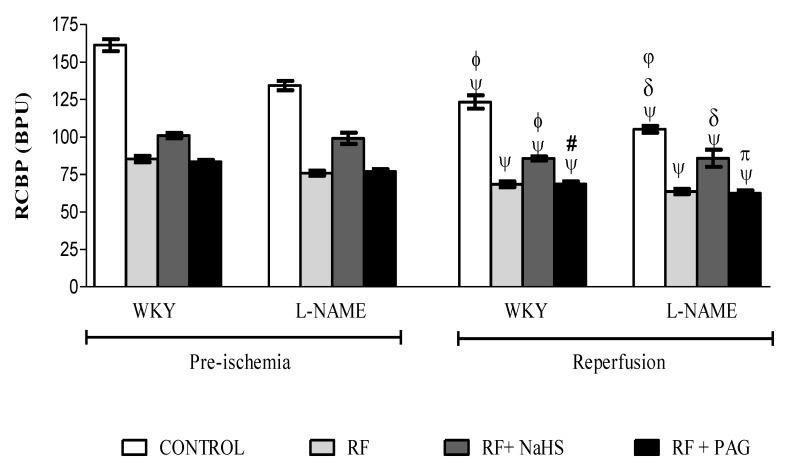
RCBP measurement in pre-ischemia and reperfusion phases (n = 6). All the data is presented as mean ± SD with significance set at 95% confidence. ψ vs. pre-ischemic phase of respective groups; ϕ vs. WKY-RF in reperfusion phase; # vs. WKY-RF+NaHS in reperfusion phase; δ vs. L-NAME-RF in reperfusion phase. π vs. L-NAME-RF+NaHS in reperfusion phase; φ vs. WKY-CONTROL in reperfusion phase.

A significant reduction (all *p* < 0.05) in renal cortical blood perfusion (RCBP) was observed in the reperfusion phase with approximately 23% in WKY-CONTROL, 19% in WKY-RF, 15% in WKY-RF+NaHS, 17% in WKY-RF+PAG, 21% in L-NAME-CONTROL, 16% in L-NAME-RF, 13% in L-NAME-RF+NaHS and 18% in L-NAME-RF+PAG when compared to their corresponding groups in the pre-ischemia phase (Figure 2). However, prophylactic treatment with NaHS caused a 25% increase in RCBP in WKY-RF+NaHS when compared to WKY-RF and a 19% increase when compared to WKY-RF+PAG. Similarly, 34% augmentation of RCBP was observed in L-NAME-RF+NaHS when compared to L-NAME-RF and a 26% increase when compared to L-NAME-RF+PAG. It was observed that WKY-RF had a significant reduction (*p* < 0.05) in RCBP by 80% as compared to the WKY-CONTROL while L-NAME-RF had a significant reduction (*p* < 0.05) in RCBP by 65% when compared to L-NAME-CONTROL. It was noticed that the L-NAME-CONTROL group had significantly decreased (all *p* < 0.05) RCBP by 17% as compared to the WKY-CONTROL group.

#### 3.2.4. Renal Function Parameters in Pre-Ischemia and Reperfusion Phases

**Figure 3:**
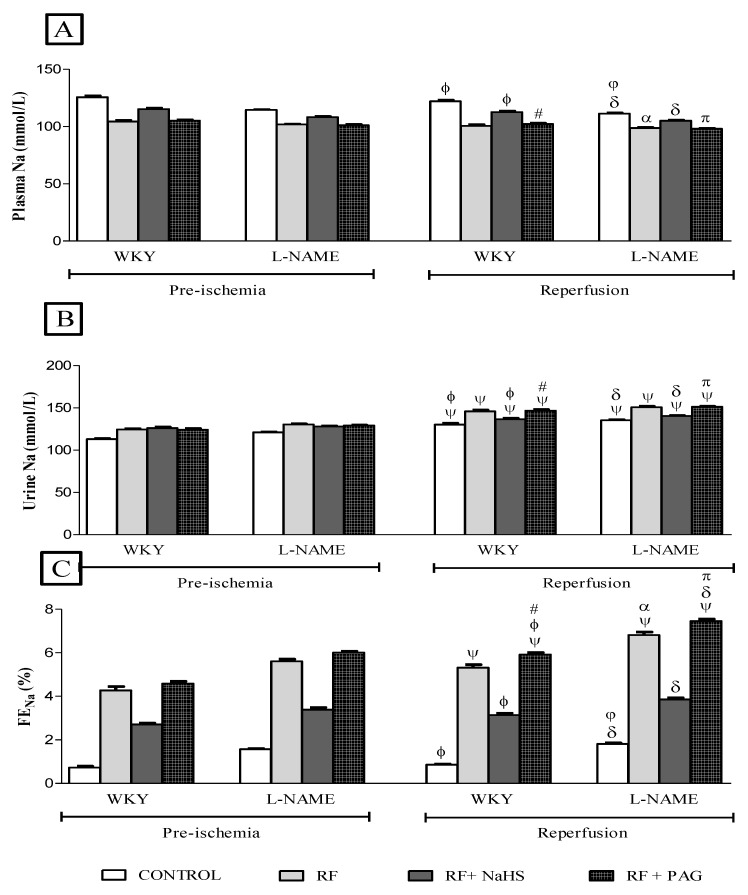
(**A**–**C**). Na in plasma, Na in urine and FENa in pre-ischemia and reperfusion phases (n = 6). (**A**): sodium in plasma, (**B**): sodium in urine, (**C**): Fractional excretion of sodium. Data was analyzed using repeated measures one-way ANOVA followed by the Bonferroni post hoc test. All the data is presented as mean ± SEM with significance set at 95% confidence (*p* < 0.05). ψ *p* < 0.05 vs. pre-ischemic phase of respective groups; ϕ *p* < 0.05 vs. WKY-RF in reperfusion phase; # *p* < 0.05 vs. WKY-RF+NaHS in reperfusion phase; δ *p* < 0.05 vs. L-NAME-RF in reperfusion phase. π *p* < 0.05 vs. L-NAME-RF+NaHS in reperfusion phase; φ *p* < 0.05 vs. WKY-CONTROL in reperfusion phase; α *p* < 0.05 vs. WKY-RF in reperfusion phase. FENa = Fractional excretion of sodium.

No significant difference was observed in plasma sodium in all groups in the reperfusion phase when compared to their corresponding groups in the pre-ischemic phase (Figure 3A). However, a significant increase (all *p* < 0.05) in urinary sodium was observed in all groups after the induction of IRI in the reperfusion phase when compared to their corresponding groups in the pre-ischemic phase (Figure 3B). Similarly, after inducing IRI, a significant increase (all *p* < 0.05) in FENa was observed in all RF groups of WKY and L-NAME rats except those groups which were pre-treated with NaHS (WKY-RF+NaHS and L-NAME-RF+NaHS) and NRF groups (WKY-CONTROL and L-NAME-CONTROL) (Figure 3C).

**Figure 4:**
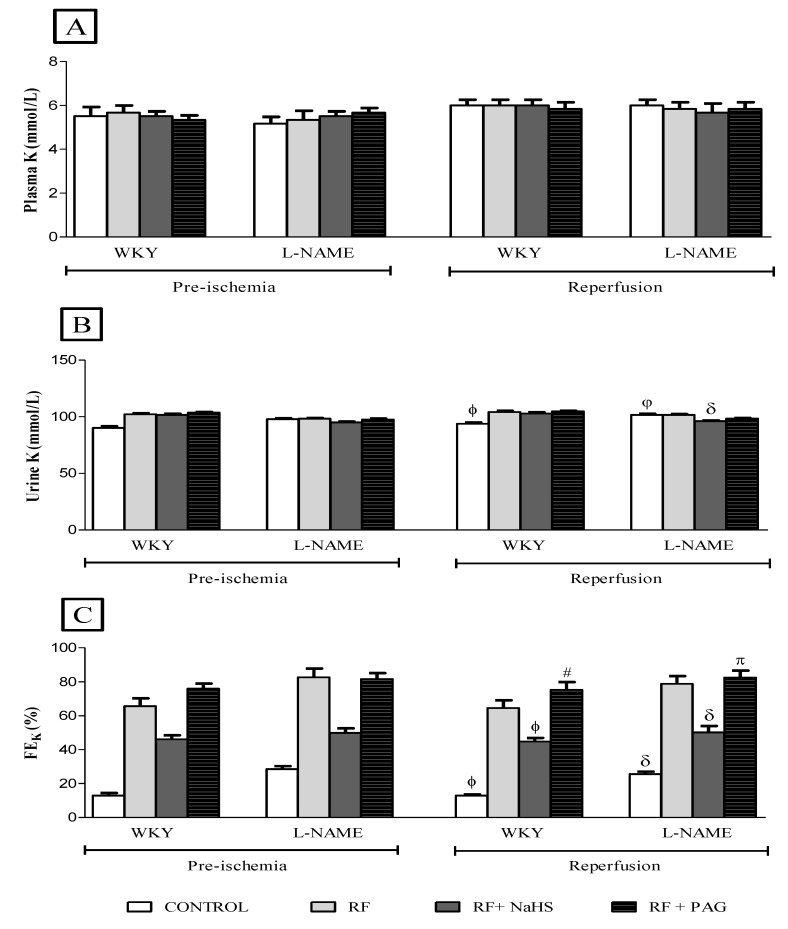
(**A**–**C**). K in plasma, K in urine and FEK in pre-ischemia and reperfusion phases (n = 6). (**A**): potassium in the plasma, (**B**): Potassium in the urine, (**C**): Fractional excretion of potassium. ϕ vs. WKY-RF in reperfusion phase; # vs. WKY-RF+NaHS in reperfusion phase; δ vs. L-NAME-RF in reperfusion phase. π vs. L-NAME-RF+NaHS in reperfusion phase; φ vs. WKY-CONTROL in reperfusion phase.

**Figure 5:**
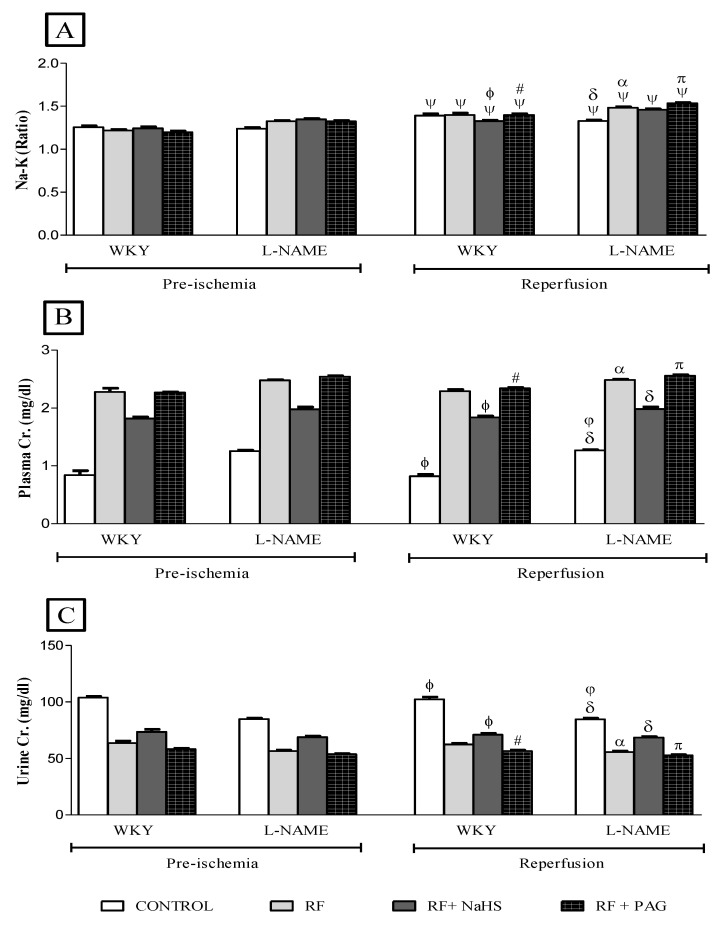
(**A**–**C**). Na:K ratio, Cr. in plasma and Cr. in urine in pre-ischemia and reperfusion phases (n = 6). (**A**): sodium to potassium ratio, (**B**): Plasma creatinine, (**C**): Urinary creatine. ψ vs. pre-ischemic phase of respective groups; ϕ vs. WKY-RF in reperfusion phase; # vs. WKY-RF+NaHS in reperfusion phase; δ vs. L-NAME-RF in reperfusion phase. π vs. L-NAME-RF+NaHS in reperfusion phase; φ vs. WKY-CONTROL in reperfusion phase; α vs. WKY-RF in reperfusion phase. Na:K ratio = Sodium:potassium ratio. Cr. = Creatinine.

No significant difference was observed in plasma potassium (Figure 4A), urinary potassium (Figure 4B) and FEK (Figure 4C) after the induction of IRI in the reperfusion phase when compared to their corresponding groups in the pre-ischemic phase. The Na:K ratio increased significantly (all *p* < 0.05) in all groups in the reperfusion phase when compared to their respective groups in pre-ischemic phase (Figure 5A). However, no significant difference was observed in plasma creatinine (Figure 5B) as well as in urinary creatinine (Figure 5C) in the reperfusion phase when compared to their corresponding groups in the pre-ischemic phase.

#### 3.2.5. Oxidative Stress Markers in Pre-Ischemia and Reperfusion Phases

**Figure 6:**
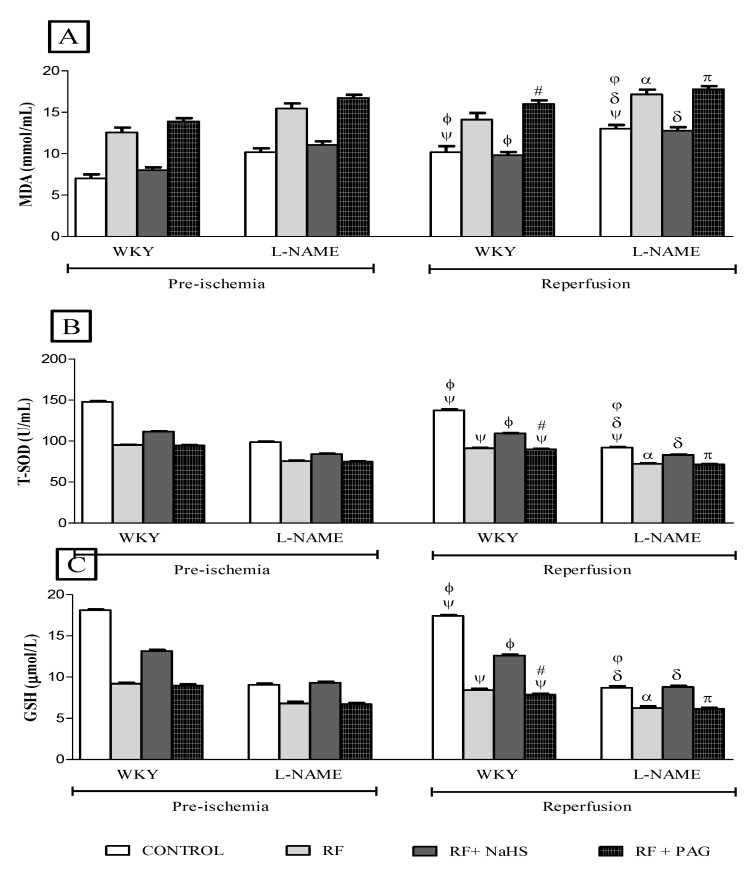
(**A**–**C**). MDA, T-SOD and GSH concentration in pre-ischemia and reperfusion phases (n = 6). (**A**): MDA levels in the plasma, (**B**): SOD levels in the plasma, (**C**): GSH levels in the plasma. ψ vs. pre-ischemic phase of respective groups; ϕ vs. WKY-RF in reperfusion phase; # vs. WKY-RF+NaHS in reperfusion phase; δ vs. L-NAME-RF in reperfusion phase. π vs. L-NAME-RF+NaHS in reperfusion phase; φ vs. WKY-CONTROL in reperfusion phase; α *p* < 0.05 vs. WKY-RF in reperfusion phase.

MDA concentration was increased significantly (*p* < 0.05) in the reperfusion phase in untreated NRF groups of WKY rats by 45% and untreated NRF groups of L-NAME rats by 28% when compared to their corresponding groups in the pre-ischemia phase. The RF groups of WKY and L-NAME treated rats did not show such a significant increase in concentration of MDA after the induction of IRI (Figure 6A). Pretreatment with NAHS caused a significant decrease (*p* < 0.05) in MDA concentration in WKY-RF+NaHS by 28% when compared to WKY-RF and a 64% decrease when compared to WKY-RF+PAG. Similarly, MDA concentration was reduced significantly in L-NAME-RF+NaHS by 25% when compared to L-NAME-RF and a 40% reduction when compared to L-NAME-RF+PAG. It was observed that WKY-RF had a significantly increased (*p* < 0.05) MDA concentration by 25% as compared to WKY-CONTROL while, L-NAME-RF had a significantly increased (*p* < 0.05) MDA concentration by 24% when compared to L-NAME-CONTROL. Similarly, L-NAME-CONTROL had a significantly high (*p* < 0.05) MDA level by 20% as compared to WKY-CONTROL. A significant reduction (all *p* < 0.05) in the T-SOD level was measured in the reperfusion phase of RF groups of WKY (WKY-RF and WKY-RF+PAG) when compared to their corresponding groups in the pre-ischemia phase except the NaHS treated group and NRF groups of WKY and L-NAME rats and RF groups of L-NAME induced hypertensive rats which did not show a significant decline in the T-SOD level after the induction of IRI (Figure 6B). It was observed that prophylactic treatment with NaHS caused a significant increase (*p* < 0.05) in the T-SOD level by 20% in WKY-RF+NaHS when compared to WKY-RF and a 17% increase when compared to WKY-RF+PAG. Similarly, NaHS treatment caused a significant increase (*p* < 0.05) in the T-SOD level by 15% in L-NAME-RF+NaHS when compared to L-NAME-RF. It was noticed that WKY-RF had a significantly reduced (*p* < 0.05) T-SOD level by 50% in comparison to WKY-CONTROL while L-NAME-RF had a significantly reduced (*p* < 0.05) T-SOD level by 27% in comparison to L-NAME-CONTROL. Similarly, L-NAME-CONTROL had a significantly reduced (all *p* < 0.05) T-SOD level by 49% when compared to WKY-CONTROL. A significant decline (*p* < 0.05) in GSH concentration was observed only in WKY-RF and WKY-RF+PAG in the reperfusion phase when compared to their corresponding groups in the pre-ischemia phase (Figure 6C). Prophylactic treatment with NaHS caused a significant increase (*p* < 0.05) in GSH concentration by 50% in WKY-RF+NaHS when compared to WKY-RF and a 37% increase when compared to WKY-RF+PAG. Similarly, NaHS treatment caused a significant increase (*p* < 0.05) in GSH concentration by 41% in L-NAME-RF+NaHS when compared to L-NAME-RF and a 30% increase when compared to L-NAME-RF+PAG. It was noticed that there was a 107% decrease in the GSH concentration of WKY-RF (*p* < 0.05) in comparison to WKY-CONTROL and a 39% decrease in the GSH concentration of L-NAME-RF (*p* < 0.05) in comparison to L-NAME-CONTROL. Similarly, L-NAME-CONTROL had a 101% decrease in (all *p* < 0.05) GSH concentration when compared WKY-CONTROL.

### 3.3. Concentration of ICAM-1 and NF-kB in Pre-Ischemia and Reperfusion Phases and Kidney Tissue

**Figure 7:**
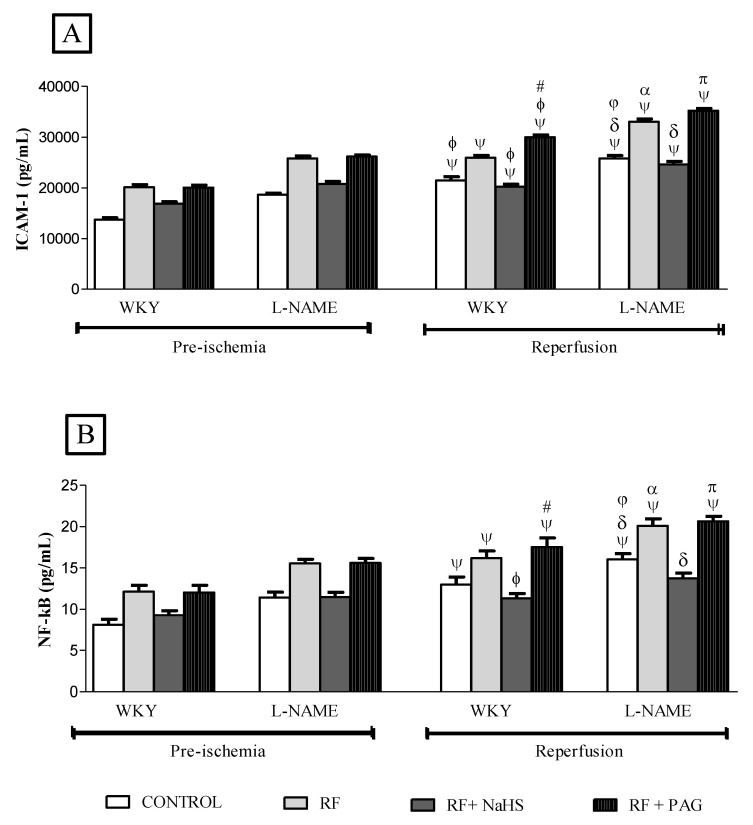

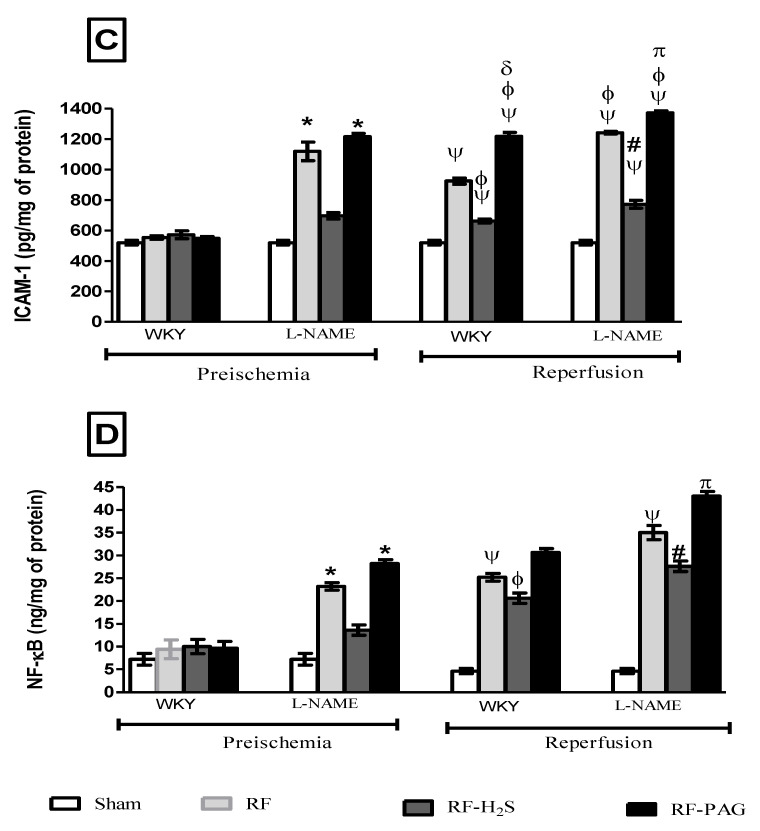
(**A**–**D**). ICAM-1 and NF-kB concentrations in the plasma and kidney tissues in pre-ischemia and reperfusion phases (n = 6). (**A**): ICAM-1 levels in the plasma, (**B**): NF-kB in the plasma, (**C**): ICAM-1 levels in the kidney tissue. (**D**): NF-kB levels in the kidney tissue. All the data is presented as mean ± SD with significance set at 95% confidence (*p* < 0.05). ψ vs. pre-ischemic phase of respective groups; ϕ vs. WKY-RF in reperfusion phase; # vs. WKY-RF+NaHS in reperfusion phase; δ vs. L-NAME-RF in reperfusion phase. π vs. L-NAME-RF+NaHS in reperfusion phase; φ vs. WKY-CONTROL in reperfusion phase; α *p* < 0.05 vs. WKY-RF in reperfusion phase, * vs. Pre-ischemic phase of respective WKY group.

A significant increase (all *p* < 0.05) in ICAM-1 concentration in the reperfusion phase was observed with approximately 56% in WKY-CONTROL, 29% in WKY-RF, 20% in WKY-RF+NaHS, 49% in WKY-RF+PAG, 38% in L-NAME-CONTROL, 28% in L-NAME-RF, 18% in L-NAME-RF+NaHS and 34% in L-NAME-RF+PAG when compared to their corresponding groups in the pre-ischemia phase (Figure 7A). However, prophylactic treatment with NaHS caused a significant decline (*p* < 0.05) in ICAM-1 concentration by 21% in WKY-RF+NaHS when compared to WKY-RF and a 48% decline in comparison to WKY-RF+PAG. Similarly, NaHS treatment caused a significant decline (*p* < 0.05) in ICAM-1 concentration by 25% in L-NAME-RF+NaHS when compared to L-NAME-RF and a 43% decline in comparison to L-NAME-RF+PAG. It was noticed that WKY-RF had a 17% increase (*p* < 0.05) in ICAM-1 concentration in comparison to WKY-CONTROL whereas, L-NAME-RF had a 21% increase (*p* < 0.05) in ICAM-1 concentration in comparison to L-NAME-CONTROL. Similarly, L-NAME-CONTROL had significantly increased (all *p* < 0.05) ICAM-1 concentration by 16% when compared WKY-CONTROL.

A significant increase (all *p* < 0.05) in NF-kB concentration was observed in the reperfusion phase with approximately 60% in WKY-CONTROL, 33% in WKY-RF, 22% in WKY-RF+NaHS, 45% in WKY-RF+PAG, 40% in L-NAME-CONTROL, 29% in L-NAME-RF, 19% in L-NAME-RF+NaHS and 32% in L-NAME-RF+PAG when compared to their corresponding groups in the pre-ischemia phase (Figure 7B). Prophylactic treatment with NaHS caused a 28% decline (*p* < 0.05) in NF-kB concentration in WKY-RF+NaHS when compared to WKY-RF and a 56% decline in comparison to WKY-RF+PAG. Similarly, NaHS treatment caused a 30% decline (*p* < 0.05) in NF-kB concentration in L-NAME-RF+NaHS when compared to L-NAME-RF and a 53% decline in comparison to L-NAME-RF+PAG. It was noticed that WKY-RF had a significantly increased (*p* < 0.05) NF-kB concentration by 17% in comparison to WKY-CONTROL while L-NAME-RF had a significantly increased NF-kB concentration by 18% in comparison to L-NAME-CONTROL. Similarly, L-NAME-CONTROL had significantly increased (all *p* < 0.05) ICAM-1 concentration by 17% when compared WKY-CONTROL.

Kidney tissue levels of both ICAM-1 and NF-kB were similar to the findings observed in plasma. Values of both ICAM-1 and NF-kB in the kidney of RF and RF-PAG groups were significantly increased (*p* < 0.05) in the preischemic and reperfusion phases, respectively, when compared to control WKY of their corresponding phases as shown in Figure 7C,D. However, treatment with H_2_S to RF-WKY significantly reduced (*p* < 0.05) the levels of ICAM-1 and NF-kB in the kidney when compared to RF-WKY groups as presented in Figure 7C,D.

### 3.4. Histopathology of Kidney Tissue by Using Hematoxyllin and Eosin Staining

**Figure 8:**
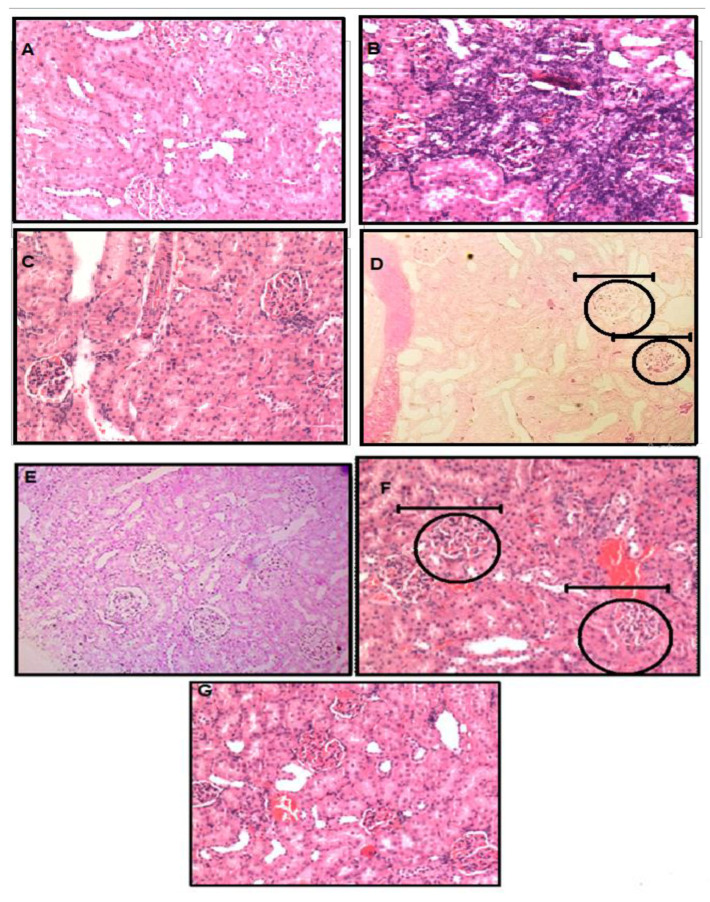
Histopathology of kidney tissue by using H&E staining. (**A)**; WKY-CONTROL; (**B**): WKY-RF; (**C**): WKY-RF+NaHS; (**D**): WKY-RF+PAG; (**E**): L-NAME-CONTROL; (**F**): L-NAME-RF; (**G**): L-NAME-RF+H2S. (**D**,**F**) round circles represents damage of the glomerulus of the kidney of WKY-RF+PAG and groups L-NAME-CONTROL. Glomerulus function is stored in (**G**) after treatment with H_2_S in L-NAME-RF+H^2^S group. Round circles and scale bars focusing glomerulus.

Glomerular apparatus was deformed characterized by thickening of Bowmen’s capsules and interstitial fibrosis was also observed in WKY-RF rats as shown in Figure 8B. Possible striped interstitial fibrosis was also observed in the WKY-RF group. All the structural changes such as the glomerulus shape and geometry were reversed when the RF group was treated with H_2_S as shown in Figure 8G.

## 4. Discussion

The present study was designed to investigate the effect exogenous H_2_S treatment on ARF as well as on renal IRI in normal and hypertensive rats (L-NAME induced). The study also hypothesized that administration of H_2_S will inhibit the concentration of NF-kB which in response will down regulate ICAM-1 concentration by its anti-inflammatory effect. Four major findings were deduced from the present study: (1) Exogenous administration of H_2_S reduced systemic hemodynamic parameters such as SBP, DBP and MAP in L-NAME-induced hypertensive rats, (2) Administration of H_2_S attenuated cisplatin induced ARF in both normotensive and L-NAME induced hypertensive rats as evidenced by improved renal functional parameters in ARF groups, (3) Administration of H_2_S reduced the severity of IRI by attenuating the concentration of pro-oxidant as evidenced by MDA and by improving the levels of antioxidant markers such as GSH and T-SOD which in response inhibited the activation of NF-kB by scavenging ROS, and (4) NF-kB inhibition by H_2_S in response down regulated the concentration of ICAM-1 due to its anti-inflammatory effect.

Oxidative stress has been documented as a key factor in the pathogenesis of cisplatin induced ARF [48], in L-NAME-induced hypertension [49] and also in injury induced by ischemia-reperfusion [50]. Similarly, inflammation is also a key contributor in cisplatin induced ARF [51], in L-NAME induced hypertension [52] and also in IRI which involves neutrophils infiltration [53]. The neutrophils infiltration further extends the injury induced by ischemia-reperfusion by producing more ROS [54]. Reperfused blood causes excessive ROS production as they are the key contributors in the sequence of injury induced by ischemia-reperfusion [55,56] by up regulating the ICAM-1 expression through the activation of NF-kB [57].

In the present study, ARF groups showed a decrease in body weight which is well in agreement to earlier reported studies [38,39,58] and it is due to toxic effects of cisplatin on GIT which causes indigestion of food [59] as evidenced by diarrhea [60]. Cisplatin also impairs the water reabsorption capacity of renal tubules due to renal tubular damage which in response causes dehydration due to loss of tubular cells and the ultimate result is the loss of body weight [61]. The increased urine output (UOP) and urine flow rate (UFR) in cisplatin induced ARF rats is due to renal tubular damage caused by cisplatin either to a thick ascending limb or collecting duct or both [62] which resulted in polyuria along with increased urinary sodium excretion. The decline in UOP and UFR following NaHS administration in cisplatin renal failure is due to its beneficial effects of H_2_S by improving tubular damage [63].

L-NAME administration caused an increase in SBP, DBP and MAP which is in line with previously reported studies [64,65]. Increase in blood pressure by L-NAME can be assumed due to its potency to remove and inactivate NO by inhibiting eNOS and also due to stimulation of sympathetic nervous system by inhibiting nNOS [66]. Decline in SBP, DBP and MAP in L-NAME induced hypertensive rats following administration of NaHS is due to its vasodilating properties [24,67]. This anti-hypertensive effect of H_2_S is in accord with previously published data [26]. Oxidative stress and increased sympathetic activity are the causative factors for increased HR in hypertensive animal models induced by L-NAME which is well in agreement to previously reported study [65].

Increase in FE_Na_ and FE_K_ in cisplatin induced ARF groups is due to injury in tubular epithelial cells of nephron and as a result of this tubular damage the reabsorptive capacity of electrolytes by renal tubules has been impaired [38]. Cisplatin is a causative agent for the structural changes in the S3 segment of proximal convoluted tubules and thus the absorptive capacity of sodium and potassium in these tubules is affected which cause increase in FE_Na_ and FE_K_ [62,68,69]. In the present study, elevated FE_Na_ and FE_K_ in hypertensive groups induced by L-NAME is coherent to previously published data [70]. L-NAME administration cause glomerulus injury and impair the handling capacity of sodium and potassium by renal tubules due to chronic inhibition of nitric oxide synthase (NOS) [71]. Reduction in FE_Na_ and FE_K_ following NaHS administration is in accord to earlier reported data [63]. Increased creatinine in plasma and decreased creatinine in urine along with a decline in creatinine clearance indicate that renal excretory function has been impaired [72] and these findings are in line with previously reported data in ARF rats [38,47,58,73] and in L-NAME induced hypertensive animals model [74]. Decrease in plasma creatinine and increase in urinary creatinine was observed in the present study following NaHS administration in RF groups of WKY and L-NAME treated rats. Decline in plasma H_2_S concentration after the induction of ARF is due to downregulation of CSE gene expression [75]. Similarly, L-NAME has also been reported to inhibit gene expression of CSE and a result of this inhibition endogenous production of H_2_S reduces [26]. In the present study, prophylactic treatment with NaHS for 5 weeks increased endogenous plasma concentration of H_2_S significantly in WKY and L-NAME induced hypertensive RF groups. Reduction in RCBP was observed in the NRF and RF groups of WKY and L-NAME induced hypertensive rats after renal IRI. The finding of the current study is in accordance with previously published studies which have evidenced a decline in renal blood flow after renal IRI [45] and indicates compromised blood perfusion in the kidney [20] due to decreased renal blood flow [76]. Vasoconstriction has been reported to be the major reason for the declined renal blood flow in renal insufficiency [77]. In the present study, RCBP was lower in the L-NAME groups as compared to normotensive WKY rats. This indicates that hypertensive rats are characterized by increased renal vasoconstriction along with vasculature resistance. This statement is well in agreement with already published data [46]. The increased RCBP in WKY and L-NAME groups following NaHS administration is due to the vasodilatory potential of H_2_S [68]. Increased preglomerular arterial vasodilation and enhanced renal blood flow by H_2_S has also been reported [78].

Increased kidney index after renal IRI is consistent with the previously published studies which stated that increased kidney weight is due to reflecting oedema and obstruction of peritubular capillaries due to infiltration of neutrophils and plugging of leukocytes at the reperfusion injury site after renal ischemia [79]. Increased kidney weight in cisplatin induced ARF rats is due to nephrotoxic effects of this drug. Cisplatin has been documented to induce different changes such as swelling and vacuolation of the lining of the glomerular endothelium, blood vessel dilation, hemorrhage in the renal cortex and renal tubule degeneration and thus due to these toxic effects kidney weight increases [80]. Increased kidney weight in L-NAME treated rats in present study is incoherence with earlier reported findings [81]. However, in the present study such increase in kidney index following renal IRI was not observed in NaHS pre-treated groups which indicate reno-protective effects of H_2_S by reducing the severity of renal injury induced by the cisplatin. In the present study, increased urinary sodium after renal IRI is in line with previous findings which have reported that IRI causes distal nephron dysfunction and impaired function of the proximal tubules are the causative factors for the profound loss of sodium in urine after renal IRI [82]. It was interesting to know that FE_Na_ which was already increased after the induction of ARF was further elevated after the induction of renal IRI. The present findings are well consistent with previously reported studies [45,83]. Interpretation of this finding is that impaired absorption capacity of epithelial cells and tubular damage were deteriorated further after renal IRI. NaHS pre-treated groups did not show such incline in FE_Na_ after renal IRI. This is due to the protective effect of NaHS which has the potency to improve electrolytes handling capacity and has been reported to reduce the severity of proximal tubular damage [63].

Renal IRI is characterized by a complex of different pathophysiological processes such as oxidative stress induced free radicals production [84]. This causes renal injury which is marked by lipid peroxidation of polyunsaturated fatty acids [85]. In present study renal IRI did not affect the already elevated MDA concentration in cisplatin induced ARF groups. However, MDA concentration was increased in NRF groups after renal IRI. This finding is well in agreement with those reported previously [86,87,88]. Increased MDA concentration in L-NAME induced hypertensive and ARF models is in line with previous findings which have reported an increase in MDA concentration in L-NAME treated [89,90] and ARF rats [91,92,93]. An increased level of MDA identifies the occurrence of oxidative stress in ARF [48] and L-NAME induced hypertensive rats [37] which support the present findings. Decline in MDA level was observed in WKY, and L-NAME treated groups following NaHS administration which is due to the antioxidant potential of H_2_S which causes scavenging of ROS and thus reduces the magnitude of oxidative stress. The present study reported a decline in the T-SOD level after renal IRI in the L-NAME and ARF groups which is in line with earlier findings [41,94,95]. Improved T-SOD level following NaHS administration is due to its potency to scavenge peroxynitrite [96] and superoxide anion [97]. GSH concentration was also lower in ARF and L-NAME induced hypertension models which is in line as reported earlier [98]. Cisplatin has been reported to cause critical depletion of GSH stored in the renal cortical cells. Thus, the insufficient concentration of GSH renders the kidney more susceptible to the nephrotoxic effects of cisplatin [99]. Reduced GSH concentration in hypertensive rats induced by L-NAME in present study is supported by previous studies [41]. An increase in GSH concentration following H_2_S administration in the present study is well consistent with previous published studies which have reported that H_2_S promotes the GSH concentration due to its antioxidant potential [99,100,101]. In the present study, administration of PAG did not change the oxidative stress markers in ARF rats following renal IRI because oxidative stress markers such as MDA concentration was already elevated and T-SOD and GSH levels were reduced in ARF groups. No further incline in MDA concentration and a decline in T-SOD and GSH levels were observed following PAG administration.

Key findings of the present study were the levels of NF-kB and ICAM-1 following renal IRI. Massive accumulation, adhesion, rolling and transmigration of leukocytes occurs in IRI [102,103]. This statement is well supported by the increased concentration of ICAM-1 in present model of renal IRI because ICAM-1 serves as a source for the leukocytes’ adhesion, rolling and their infiltration. Upregulated expression of ICAM-1 indicates that the process of inflammation is involved in renal IRI. Increased NF-kB concentration following renal IRI further strengthened the notion that upregulation of ICAM-1 depends upon NF-kB activation as evidenced in the present study. It was observed that ARF and L-NAME induced hypertensive rats exhibited increased concentration of ICAM-1 and NF-kB as compared to normotensive WKY rats. This indicates that L-NAME induced hypertensive and ARF rats are more prone to the consequences of inflammation and oxidative stress as compared to normotensive NRF rats following renal IRI. In the present study, chronic administration of NaHS reduced the concentration of ICAM-1 and NF-kB after renal IRI. The decline of endogenous concentration of H_2_S by administration of PAG increased the concentration of ICAM-1 and NF-kB. The present findings further strengthened the perception that the reperfused kidney is more render to the extent of IRI when the endogenous concentration of H_2_S is reduced and enhancing the physiological concentration of H_2_S reduces the intensity of IRI by potentiating its anti-inflammatory and antioxidant potential as evidenced in the present study by declined ICAM-1 and NF-kB concentration.

**Figure 9:**
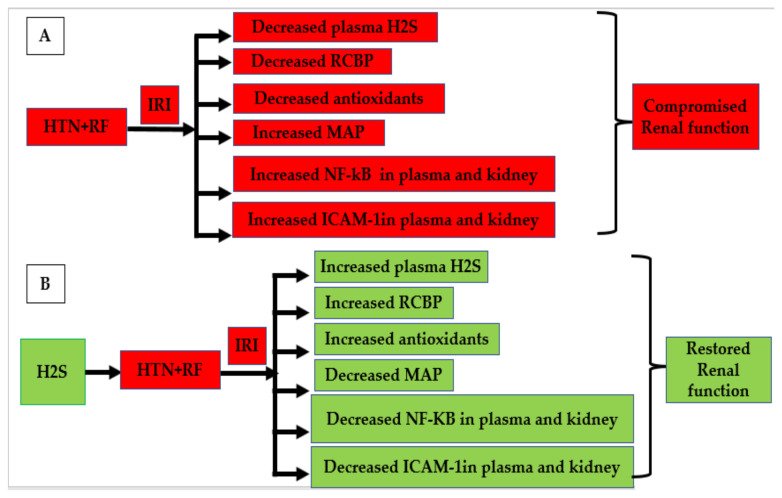
Summary of parameters changed during renal ischemia-reperfusion injury (IRI) in hypertensive (HTN) renal failure (RF) cases which impaired the performance of the kidney as shown in (**A**) and prophylactic treatment of H_2_S on all parameters during renal ischemia-reperfusion injury (IRI) in hypertensive (HTN) renal failure (RF) cases reverse all the parameters of Figure 9A and restored the performance of the kidney as shown in (**B**).

ROS are the inducers for the activation of NF-kB. Thus, it can be deduced from the findings of the present study that H_2_S has the ability to scavenge ROS due to its antioxidant potential as reported by previous findings [104]. Thus, scavenging of ROS in response inhibited the activation of NF-kB following NaHS administration. Once NF-kB was inhibited, it in response down regulated the concentration of ICAM-1. The findings of the present study confirmed that H_2_S has both antioxidant potential as well as anti-inflammatory properties. Discussion can be summarized that exogenous administration of H_2_S donor in renal failure hypertensive rats protect kidney from renal ischemia-reperfusion injury by increasing antioxidant activities systemically which in turn reduces levels of ICAM-1 and NF-kB concentration globally and more specifically in the kidney resulting in improved renal excretory functions and increased renal cortical blood perfusion as shown in Figure 9A,B.

## 5. Conclusions

These findings demonstrate that H_2_S not only reduced the severity of cisplatin induced ARF but also reduced the severity of renal IRI by upregulating antioxidants along with the reduction of NF-kB and ICAM-1 in normotensive WKY and L-NAME induced hypertensive rats. The findings of the present study demonstrate that administration of H_2_S can be a beneficial prophylactic treatment approach which can provide renal protection in renal transplantations by reducing ischemia reperfusion injury.

## Figures and Tables

**Table 1:** Physiological data during metabolic collections on days 0, 21st and 35th of the study.

Parameters	Names of Groups	Day 0	Day 21	Day 35
Body Weight	WKY-CONTROL	236 ± 2	272 ± 4 ψ	316.0 ± 8.9 ψ ϕ δ
	WKY-RF	240 ± 6	277 ± 10.0 ψ	282.5 ± 17.2 ψ
	WKY-RF+NaHS	236 ± 5	279 ± 12 ψ	276.8 ± 6.4 ψ
	WKY-RF+PAG	231 ± 6	272 ± 7 ψ	267 ± 5 ψ
	L-NAME-CONTROL	233 ± 5	260.7 ± 4.1 ψ	284 ± 6 ψ ϕ φ π
	L-NAME-RF	232 ± 4	262.2 ± 4.2 ψ	259 ± 7 ψ δ
	L-NAME-RF+NaHS	228 ± 5	266.5 ± 2.2 ψ	273 ± 3ψ φ
	L-NAME-RF+PAG	234 ± 6	260 ± 5 ψ	261 ± 8 ψ
Water Intake	WKY-CONTROL	50± 6	51.3 ± 5.6	51.6 ± 6.1
	WKY-RF	43 ± 5	42 ± 6	56 ± 11
	WKY-RF+NaHS	44 ± 5	46.6 ± 3.0	52 ± 7
	WKY-RF+PAG	41 ± 8	39.6 ± 5.9	53 ± 6
	L-NAME-CONTROL	42 ± 5	47.0 ± 4.8	52.5 ± 9
	L-NAME-RF	43 ± 5	42.1 ± 5.5	56 ± 11
	L-NAME-RF+NaHS	43 ± 6	42.5 ± 4.7	48 ±5
	L-NAME-RF+PAG	45 ±7	47.3 ± 4.9	52 ± 19
Urinary Out Put	WKY-CONTROL	21 ± 3	21 ± 3	20 ± 4
	WKY-RF	16 ± 2	16 ± 2	31 ± 8 ψ ϕ
	WKY-RF+NaHS	16 ± 2	16 ± 5	25 ± 3 ϕ
	WKY-RF+PAG	21 ± 4	19 ± 3	28 ± 5
	L-NAME-CONTROL	19.8 ± 6.9	17 ± 8	19 ± 8 φ
	L-NAME-RF	14.8 ± 2.1	18± 3	28.6 ± 6.2 ψ ϕ
	L-NAME-RF+NaHS	14 ± 2	14± 2	19 ± 2 φ
	L-NAME-RF+PAG	14.1 ± 1.4	17.6 ± 2.4	28 ± 4 ψ ϕ
Urine flow rate	WKY-CONTROL	6 ± 0.8	5 ± 0.7	4 ± 0.7 δ
	WKY-RF	5 ± 0.6	4 ± 0.5	8 ± 3 ψ ϕ
	WKY-RF+NaHS	5 ± 0.5	4 ± 1.0	6± 0.8
	WKY-RF+PAG	6.24 ± 1.5	4.90 ± 0.8	7 ± 1
	L-NAME-CONTROL	6 ± 2	4 ± 2	5 ± 2 φ
	L-NAME-RF	4 ± 0.6	5 ± 0.7	8 ± 2 ψ ϕ
	L-NAME-RF+NaHS	4 ± 0.7	4 ± 0.5	5 ± 0.5 φ
	L-NAME-RF+PAG	4 ± 0.3	5 ± 0.5	7 ± 1 ψ ϕ

All the data is presented as mean ± SEM and was analyzed by using repeated measures one-way ANOVA followed by the Bonferroni post hoc test with significance set at 95% confidence (*p* < 0.05). ψ *p* < 0.05 vs. day 0 of respective groups; ϕ *p* < 0.05 vs. 21st day of respective groups; δ *p* < 0.05 vs. WKY-RF on 35th day; φ *p* < 0.05 vs. L-NAME-RF on 35th day; π *p* < 0.05 vs. WKY-CONTROL on 35th day.

**Table 2:** Systemic hemodynamics data on days 0, 21st and 35th of the study.

Parameters	Groups	Day 0	Day 21	Day 35
Systolic blood pressure (SBP)	WKY-CONTROL	119 ± 7	121 ± 1	118 ± 4
	WKY-RF	115 ± 3	119 ± 4	116 ± 4
	WKY-RF+NaHS	118 ± 4	116 ± 2	116 ± 3
	WKY-RF+PAG	119 ± 3	116.5 ± 3.2	118.2 ± 2.2
	L-NAME-CONTROL	121 ± 3	174 ± 10 ψ	200 ± 12 ψ ϕ π
	L-NAME-RF	119 ± 4	162 ± 6 ψ	193 ± 6 ψ ϕ δ
	L-NAME-RF+NaHS	117 ± 3	139 ± 3 ψ	176 ± 70 ψ ϕ φ
	L-NAME-RF+PAG	115 ± 5	159 ± 4 ψ	196 ± 7 ψ ϕ
Diastolic blood pressure (DBP)	WKY-CONTROL	89 ± 5	88 ± 2	89 ± 1
	WKY-RF	89± 2	89 ± 2	89 ± 2
	WKY-RF+NaHS	91 ± 2	89 ± 3	88 ± 2
	WKY-RF+PAG	88 ± 1	88 ± 2	89 ± 1
	L-NAME-CONTROL	91 ± 2	128 ± 6 ψ	156 ± 6 ψ ϕ π
	L-NAME-RF	90 ± 2	133 ± 4 ψ	159 ± 5 ψ ϕ δ
	L-NAME-RF+NaHS	88 ± 1	110 ± 2 ψ	132 ± 4 ψ ϕ φ
	L-NAME-RF+PAG	88 ± 2	128 ± 12 ψ	159 ± 4 ψ ϕ
Mean arterial pressure (MAP)	WKY-CONTROL	99 ± 4	99 ± 1	98 ± 2
	WKY-RF	97 ± 2	99 ± 0.5	97 ± 1
	WKY-RF+NaHS	99 ± 1	98 ± 2	96 ± 2
	WKY-RF+PAG	98± 1.0	98 ± 1	9.8 ± 1
	L-NAME-CONTROL	101 ± 2	143 ± 3 ψ	171 ± 5 ψ ϕ π
	L-NAME-RF	99 ± 2	142 ± 4 ψ	172 ± 4 ψ ϕ δ
	L-NAME-RF+NaHS	98± 1	119 ± 2 ψ	146.0 ± 4 ψ ϕ φ
	L-NAME-RF+PAG	97 ± 2	141 ± ψ	173 ± 4 ψ ϕ
Heart rate (HR)	WKY-CONTROL	325 ± 10	322.7 ± 15	309 ± 14
	WKY-RF	316± 9	322 ± 10	308 ± 10
	WKY-RF+NaHS	321± 30	303 ± 18	320 ± 33
	WKY-RF+PAG	315± 8	304 ± 16	306 ± 16
	L-NAME-CONTROL	314± 11	371 ± 12 ψ	412 ± 14 ψ ϕ π
	L-NAME-RF	313 ± 14	373 ± 11 ψ	419 ± 23ψ ϕ δ
	L-NAME-RF+NaHS	314 ± 7	332 ± 9	402 ± 16 ψ ϕ
	L-NAME-RF+PAG	302 ± 18	385 ± 5 ψ	428 ± 20 ψ ϕ

All the data is presented as mean ± SEM and was analyzed by using repeated measures one-way ANOVA followed by the Bonferroni post hoc test with significance set at 95% confidence. ψ *p* < 0.05 vs. day 0 same groups; ϕ vs. day 21 of same groups; δ *p* < 0.05 vs. WKY-RF on day 35; φ vs. L-NAME-RF on day 35; π vs. WKY-CONTROL on day 35.

**Table 3:** Renal function parameters on days 0, 21st and 35th of the study.

Parameters	Groups	Day 0	Day 21	Day 35
Sodium in plasma	WKY-CONTROL	129 ± 1.9	131 ± 2.3	129 ± 3.4 δ
	WKY-RF	122 ± 3.3	125 ± 2.8	105 ± 2.8 ψ ϕ
	WKY-RF+NaHS	131 ± 2.6	129 ± 2.4	114 ± 2.6 ψ ϕ δ
	WKY-RF+PAG	125 ± 3.8	127 ± 3.0	104 ± 2.9 ψ ϕ
	L-NAME-CONTROL	127 ± 1.4	121 ± 1.4 ψ	115 ± 1.4 ψ ϕ φ π
	L-NAME-RF	128 ± 1.5	122 ± 1.7 ψ	102 ± 1.9 ψ ϕ
	L-NAME-RF+NaHS	125 ± 1.4	121 ± 1.5	107 ± 1.4 ψ ϕ
	L-NAME-RF+PAG	129 ± 1.2	123 ± 1.3 ψ	100 ± 1.0 ψ ϕ
Potassium in urine	WKY-CONTROL	112 ± 2.0	113 ± 2.2	112 ± 2.4 δ
	WKY-RF	111 ± 2.0	113 ± 2.0	124 ± 3.7 ψ ϕ
	WKY-RF+NaHS	114 ± 2.6	112 ± 2.4	125 ± 3.8 ψ ϕ
	WKY-RF+PAG	114 ± 2	112 ± 2.2	125 ± 4.1 ψ ϕ
	L-NAME-CONTROL	114.2 ± 1.4	118 ± 1.5	122 ± 1.6 ψ φ π
	L-NAME-RF	114 ± 1.0	118 ± 1.3	129 ± 1.7 ψ ϕ δ
	L-NAME-RF+NaHS	113 ± 1.6	117 ± 1.3	126 ± 2.1 ψ ϕ
	L-NAME-RF+PAG	112 ± 1.2	116 ± 1.4	130 ± 1.7 ψ ϕ
Potassium in plasma	WKY-CONTROL	5.33 ± 0.5	5.66 ± 1.2	5.50 ± 1.0
	WKY-RF	5.16 ± 0.7	5.50 ± 0.8	5.66 ± 0.8
	WKY-RF+NaHS	5.33 ± 1.0	5.50 ± 0.5	5.33 ± 0.5
	WKY-RF+PAG	5.50 ± 0.8	5.83 ± 0.7	5.33 ± 0.5
	L-NAME-CONTROL	5.66 ± 0.8	5.33 ± 1.0	5.50 ± 1.0
	L-NAME-RF	5.66 ± 0.8	5.83 ± 0.9	5.66 ± 0.8
	L-NAME-RF+NaHS	5.33 ± 1.0	5.50 ± 0.5	5.16 ± 0.7
	L-NAME-RF+PAG	5.33 ± 0.5	5.83 ± 0.7	5.50 ± 1.0
Potassium in urine	WKY-CONTROL	88.0 ± 1.4	90.3 ± 1.7	88.3 ± 2.5 δ
	WKY-RF	81.5 ± 2.4	83.6 ± 2.1	103.3 ± 2.1 ψ ϕ
	WKY-RF+NaHS	79.6 ± 2.5	82.6 ± 2.5	99.6 ± 2.5 ψ ϕ
	WKY-RF+PAG	84.3 ± 1.8	87.5 ± 1.8	105.5 ± 1.8 ψ ϕ
	L-NAME-CONTROL	87.0 ± 1.4	92.3 ± 1.5 ψ	98.5 ± 1.5 ψ ϕ π
	L-NAME-RF	85.8 ± 1.1	91.8 ± 1.1 ψ	97.6 ± 1.3 ψ ϕ δ
	L-NAME-RF+NaHS	83.5 ± 1.8	87.0 ± 1.4	93.1 ± 1.6 ψ ϕ φ
	L-NAME-RF+PAG	86.1 ± 1.1	92.0 ± 2.0 ψ	98.0 ± 3.0 ψ ϕ
Fractional excretion of sodium (FE_Na)_	WKY-CONTROL	0.68 ± 0.04	0.62 ± 0.09	0.70 ± 0.15 δ
	WKY-RF	0.73 ± 0.07	0.69 ± 0.12	4.25 ± 0.34 ψ ϕ
	WKY-RF+NaHS	0.64 ± 0.09	0.61 ± 0.17	2.76 ± 0.18 ψ ϕ δ
	WKY-RF+PAG	0.66 ± 0.02	0.64 ± 0.01	4.44 ± 0.29 ψ ϕ
	L-NAME-CONTROL	0.69 ± 0.01	0.97 ± 0.03	1.55 ± 0.04 ψ ϕ φπ
	L-NAME-RF	0.68 ± 0.04	1.01 ± 0.04	5.78 ± 0.32 ψ ϕ δ
	L-NAME-RF+NaHS	0.69 ± 0.06	0.91 ± 0.06	3.45 ± 0.15 ψ ϕ φ
	L-NAME-RF+PAG	0.66 ± 0.05	0.97 ± 0.03	6.18 ± 0.29 ψ ϕ
Fractional excretion of potassium (FE_K_)	WKY-CONTROL	12.8 ± 1.1	12.7 ± 4.7	13.3 ± 2.8 δ
	WKY-RF	13.1 ± 3.2	11.7 ± 1.2	66.4 ± 7.5 ψ ϕ
	WKY-RF+NaHS	11.3 ± 2.8	10.5 ± 2.4	47.5 ± 6.2 ψ ϕ δ
	WKY-RF+PAG	11.2 ± 1.3	11.1 ± 1.4	73.2 ± 5.7 ψ ϕ
	L-NAME-CONTROL	12.5 ± 1.0	17.6 ± 2.8	26.4 ± 4.5 ψ φ π
	L-NAME-RF	11.8 ± 1.8	16.8 ± 2.8	80.1 ± 10.9 ψ ϕ δ
	L-NAME-RF+NaHS	12.4 ± 2.8	15.1 ± 2.1	53.3 ± 6.3 ψ ϕ φ
	L-NAME-RF+PAG	12.3 ± 1.4	17.4 ± 2.3	87.2 ± 17.2 ψ ϕ
Urinary sodium volume (U_Na_V)	WKY-CONTROL	0.096 ± 0.013	0.098 ± 0.013	0.095 ± 0.016 δ
	WKY-RF	0.075 ± 0.011	0.074 ± 0.009	0.161 ± 0.046 ψ ϕ
	WKY-RF+NaHS	0.075 ± 0.010	0.072 ± 0.021	0.130 ± 0.020 ψ ϕ
	WKY-RF+PAG	0.098 ± 0.023	0.090 ± 0.014	0.147 ± 0.028 ψ ϕ
	L-NAME-CONTROL	0.094 ± 0.033	0.083 ± 0.037	0.095 ± 0.040 φ
	L-NAME-RF	0.070 ± 0.009	0.087 ± 0.013	0.154 ± 0.032 ψϕ
	L-NAME-RF+NaHS	0.066 ± 0.011	0.066 ± 0.010	0.098 ± 0.011 φ
	L-NAME-RF+PAG	0.066 ± 0.006	0.085 ± 0.011	0.153 ± 0.026 ψ ϕ
Urinary potassium volume (U_k_V)	WKY-CONTROL	0.104 ± 0.017	0.105 ± 0.016	0.107 ± 0.021
	WKY-RF	0.122 ± 0.021	0.129 ± 0.015	0.084 ± 0.021
	WKY-RF+NaHS	0.123 ± 0.020	0.139 ± 0.044	0.097 ± 0.014
	WKY-RF+PAG	0.103 ± 0.029	0.112 ± 0.018	0.092 ± 0.016
	L-NAME-CONTROL	0.115 ± 0.033	0.152 ± 0.057	0.145 ± 0.050 φ
	L-NAME-RF	0.141 ± 0.020	0.127 ± 0.020	0.085 ± 0.020 ψ
	L-NAME-RF+NaHS	0.146 ± 0.043	0.155 ± 0.022	0.121 ± 0.014
	L-NAME-RF+PAG	0.147 ± 0.017	0.127 ± 0.018	0.085 ± 0.015 ψ
Na:K ratio	WKY-CONTROL	1.27 ± 0.02	1.25 ± 0.02	1.27 ± 0.03
	WKY-RF	1.36 ± 0.05	1.35 ± 0.04	1.20 ± 0.04 ψ ϕ
	WKY-RF+NaHS	1.43 ± 0.04	1.35 ± 0.03	1.25 ± 0.05 ψ ϕ
	WKY-RF+PAG	1.35 ± 0.02	1.28 ± 0.04	1.18 ± 0.02 ψ ϕ
	L-NAME-CONTROL	1.31 ± 0.02	1.28 ± 0.02	1.24 ± 0.01 φ
	L-NAME-RF	1.33 ± 0.02	1.28 ± 0.01	1.32 ± 0.01 δ
	L-NAME-RF+NaHS	1.36 ± 0.04	1.35 ± 0.03	1.36 ± 0.04
	L-NAME-RF+PAG	1.30 ± 0.03	1.27 ± 0.03	1.33 ± 0.04
Creatinine In plasma	WKY-CONTROL	0.84 ± 0.03	0.74 ± 0.13	0.82 ± 0.17 δ
	WKY-RF	0.85 ± 0.10	0.83 ± 0.12	2.21 ± 0.13 ψ ϕ
	WKY-RF+NaHS	0.79 ± 0.09	0.78 ± 0.19	1.82 ± 0.08 ψ ϕ δ
	WKY-RF+PAG	0.79 ± 0.03	0.81 ± 0.04	2.24 ± 0.02 ψ ϕ
	L-NAME-CONTROL	0.85 ± 0.02	0.96 ± 0.02	1.25 ± 0.19 ψ ϕ φ π
	L-NAME-RF	0.84 ± 0.05	0.99 ± 0.04	2.50 ± 0.04 ψ ϕ δ
	L-NAME-RF+NaHS	0.81 ± 0.07	0.93 ± 0.05	1.98 ± 0.08 ψ ϕ φ
	L-NAME-RF+PAG	0.84 ± 0.05	1.01 ± 0.06	2.55 ± 0.05 ψ ϕ
Creatinine in urine	WKY-CONTROL	106.4 ± 3.8	103.6 ± 3.2	101.8 ± 3.6 δ
	WKY-RF	105.4 ± 4.3	108.2 ± 3.1	61.7 ± 4.0 ψ ϕ
	WKY-RF+NaHS	108.8 ± 5.3	111.5 ± 5.9	72.3 ± 6.2 ψ ϕ δ
	WKY-RF+PAG	109.6 ± 2.5	111.1 ± 2.9	61.0 ± 2.0 ψ ϕ
	L-NAME-CONTROL	110.7 ± 1.8	96.2 ± 3.0 ψ	85.4 ± 2.8 ψ ϕ φ π
	L-NAME-RF	110.6 ± 2.2	94.9 ± 2.8 ψ	54.6 ± 2.1 ψ ϕ δ
	L-NAME-RF+NaHS	105.9 ± 1.2	99.3 ± 1.6	68.0 ± 2.0 ψ ϕ φ
	L-NAME-RF+PAG	111.9 ± 1.9	93.4 ± 1.2 ψ	53.7 ± 1.6 ψ ϕ
Creatinine clearance	WKY-CONTROL	7.68 ± 1.38	7.62 ± 0.98	5.80 ± 0.64 δ
	WKY-RF	5.94 ± 1.25	5.26 ± 1.33	2.15 ± 0.72 ψ ϕ
	WKY-RF+NaHS	6.36 ± 0.58	5.71 ± 1.90	2.48 ± 0.45 ψ ϕ
	WKY-RF+PAG	8.58 ± 2.00	6.72 ± 1.22	1.99 ± 0.37 ψ ϕ
	L-NAME-CONTROL	6.71 ± 1.71	4.15 ± 1.89 ψ	2.92 ± 1.12 ψ π
	L-NAME-RF	5.80 ± 0.83	4.49 ± 0.85	1.68 ± 0.38 ψ ϕ
	L-NAME-RF+NaHS	5.64 ± 1.46	3.80 ± 0.78	1.63 ± 0.26 ψ
	L-NAME-RF+PAG	5.58 ± 0.68	4.33 ± 0.62	1.58 ± 0.25 ψ ϕ

Data is presented as mean ± SEM and was analyzed by using repeated measures one-way ANOVA followed by the Bonferroni post hoc test with significance set at 95% confidence (*p* < 0.05). ψ vs. day 0 of corresponding groups; ϕ vs. day 21 of respective groups; δ vs. WKY-RF on 35th day; φ *p* < 0.05 vs. L-NAME-RF on 35th day π *p* < 0.05 vs. WKY-CONTROL on day 35.

**Table 4:** Body weight, kidney weight and kidney index on acute experiment day (day 36).

Groups	BW (gm)	KW (gm)	KI (%)
WKY-CONTROL	317 ± 0.8	1.05 ± 0.05	0.3 ± 0.01
WKY-RF	282 ± 17.1 ψ	1.04 ± 0.04	0.3 ± 0.02 ψ
WKY-RF+NaHS	274 ± 6.9	0.94 ± 0.01 ϕ	0.3 ± 0.00
WKY-RF+PAG	266 ± 5.3	1.01 ± 0.08	0.3 ± 0.02
L-NAME-CONTROL	284 ± 9.0 ψ	1.08 ± 0.05	0.3 ± 0.01
L-NAME-RF	258 ± 6.8 ϕ #	1.21 ± 0.02 ϕ #	0.4 ± 0.02 ϕ #
L-NAME-RF+NaHS	273 ± 3.4	1.13 ± 0.03	0.4± 0.01
L-NAME-RF+PAG	260 ± 8.1	1.19 ± 0.03	0.4 ± 0.01 π

ψ vs. WKY-CONTROL; ϕ vs. WKY-RF; # vs. L-NAME-CONTROL; π vs. L-NAME-RF+NaHS.

## Data Availability

Data will be provided on demand.

## Supplementary Materials

The following supporting information can be downloaded at: https://www.mdpi.com/article/10.3390/life12111819/s1, Figure S1: Flow Chart of the Study Protocol.
