# Supplementary figures and images for: Prophylactic Treatment with Hydrogen Sulphide Can Prevent Renal Ischemia-Reperfusion Injury in L-NAME Induced Hypertensive Rats with Cisplatin-Induced Acute Renal Failure
Source: Life (Basel). 2022 Nov 8;12(11):1819. doi: 10.3390/life12111819 (PMC9695289; doi:10.3390/life12111819)

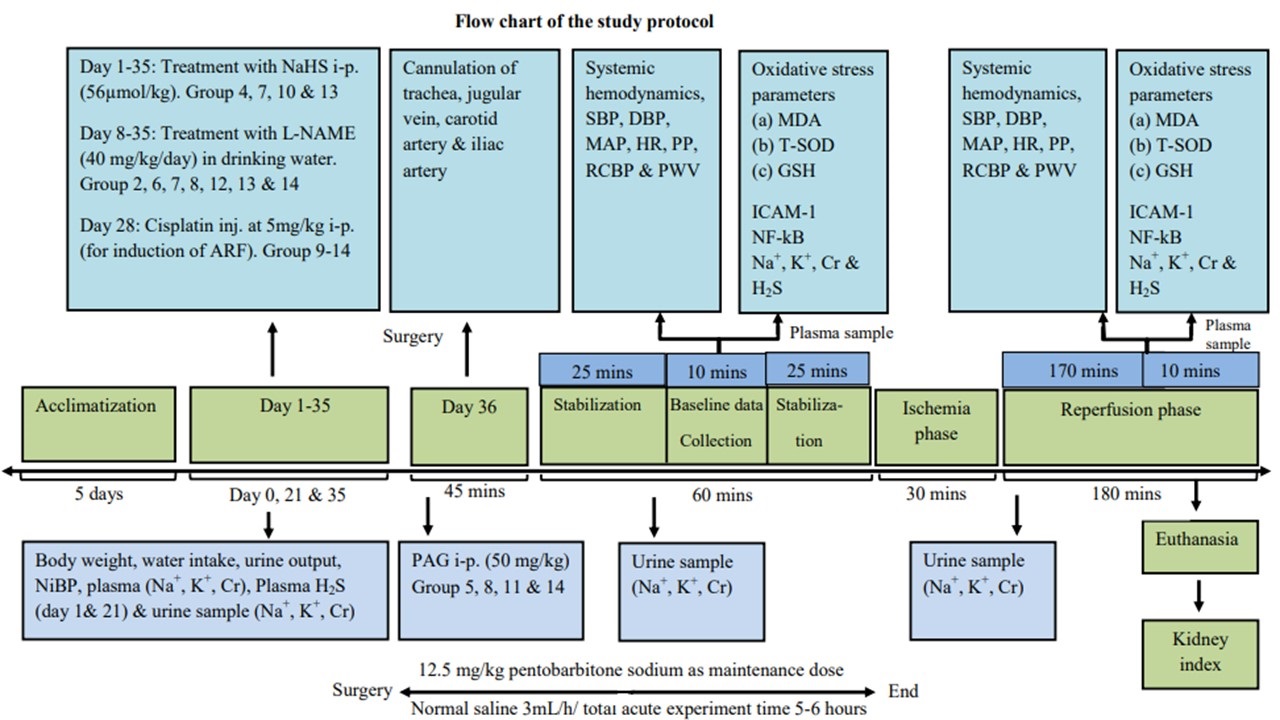

Supplement: Supplementary file 1 [file life-12-01819-s001.zip › Figure S1 - Flow Chart of the Study Protocol.jpg]
